# Use of Three-dimensional Printing for Tibial Pilon Fracture Diagnosis and Treatment

**DOI:** 10.1055/s-0044-1785514

**Published:** 2024-06-22

**Authors:** Leonardo Wustro, João Luiz Vieira da Silva, Bruno Arnaldo Bonacin Moura, Helena Squizatto Schoenberger, Debora Takito, Júlio César Honório D'Agostini

**Affiliations:** 1 Complexo Hospitalar do Trabalhador, Curitiba, PR, Brasil; 2 Complexo Hospital de Clínicas, Universidade Federal do Paraná (UFPR), Curitiba, PR, Brasil; 3 Hospital da Santa Casa de Curitiba, Curitiba, PR, Brasil

**Keywords:** fractures, bone, printing, three-dimensional, tibial fractures

## Abstract

**Objective** To evaluate whether three-dimensional (3D) printing increases agreement in the classification of tibial pilon fractures.

**Methods** Orthopedists and traumatologists reviewed radiographs, computed tomography scans with 3D reconstruction, and prototyping 3D printing, and classified the fractures based on the Rüedi-Allgöwer and Arbeitsgemeinschaft für Osteosynthesefragen (AO, Association for the Study of Internal Fixation) Foundation/Orthopedic Trauma Association (AO/OTA) classification systems. Next, data evaluation used Kappa agreement coefficients.

**Results** The use of the 3D model did not improve agreement for tibial pilon fractures regarding the treatment proposed by the groups. Regarding the classification systems, the agreement only improved concerning the AO/OTA classification when the 3D model was used in the assessment by the foot and ankle specialists.

**Conclusion** Although 3D printing is statistically relevant for surgeons specializing in foot and ankle, its values remain lower than optimal.

## Introduction

Pilon fractures represent 1% of lower limb fractures and approximately 5% to 10% of tibial fractures. 1 Furthermore, 20% to 40% of these fractures are open. 2 Pilon fractures are more common in the fourth decade of life and in male subjects .3 They often result from high-energy trauma with axial load and shear force, 4 such as in falls and traffic accidents. 5 These fractures can also occur due to rotational trauma, such as in injuries due to sporting activities. 6 In this case, the fracture results from low-energy trauma and consequently causes less damage to soft tissues and less comminution. Foot position at impact, as well as the direction and amplitude of the force account for the several fracture patterns and degrees of comminution. 7

Classification systems are helpful in the clinical practice to assess injury severity, guide surgical treatment, and facilitate communication and comparison for academic purposes. The most commonly used classifications for tibial pilon fractures include the Rüedi-Allgöwer and the Arbeitsgemeinschaft für Osteosynthesefragen (AO, Association for the Study of Internal Fixation) Foundation/Orthopedic Trauma Association (AO/OTA) classifications. The AO/OTA classification considers the extent of joint involvement and comminution, 8 dividing pilon fractures into 3 groups: 43-A (extra-articular fracture), 43-B (partial articular fracture), and 43-C (complete joint fracture) .9 These groups are divided into three subgroups each, with increasing complexity and progressively worse prognosis. 6 The Rüedi-Allgöwer classification divides fractures into three types per comminution degree and joint displacement. Type I is a simple articular fracture with no displacement, type II is a simple articular fracture with displacement of the articular surface but no comminution, and type III is a comminuted articular fracture with displacement. 10

The surgical indication for the fixation of pilon fractures includes open fractures with 2 mm of joint displacement, talar subluxation, or misalignment greater than 5°. 10 The main therapeutic goals are to protect the soft tissues, ensure adequate alignment, restore the joint surface, 3 and enable early rehabilitation and mobilization. 10

The conduct and surgical time depend on the patient's general health status, the condition of adjacent soft tissues, fracture comminution, and the surgeon's experience. 11 The literature reports several treatment options for tibial pilon fractures, including open reduction and internal fixation, external fixation, and several combinations and modifications of these techniques. 12 External fixation often occurs first to improve adjacent soft tissue damage, 13 reducing the rates of infection, dehiscence, and osteomyelitis. 12 This two-stage procedure is popular worldwide to treat tibial pilon fractures. 14 On the other hand, when the soft tissue is in good condition, open reduction and internal fixation are initially performed. 15 The general sequence to treat tibial pilon fractures includes length and alignment reestablishment, articular surface restoration, metaphyseal defect filling, and reconnection to the diaphysis. 10

Three-dimensional (3D) printing is a rapid prototyping technology that uses a 3D digital model to build an object. It has developed quickly and gained good visibility in orthopedics because the printed model enables fracture visualization and the creation of a precise, customized plan for patients. In addition, the model enables procedural simulation due to preoperative visualization of fracture anatomy and improves the communication between doctor and patient. 16

The present study aims to evaluate the agreement of tibial pilon fracture classification and proposed treatment based on imaging exams and comparison with 3D printing.

## Materials and Methods

The Ethics Committee approved the current study, which was registered on Plataforma Brasil under CAAE number 52795321.0.0000.5225.

The present observational, cross-sectional, retrospective study included 16 professionals in the field of Orthopedics and Traumatology, namely 8 resident physicians, 4 orthopedist, and 4 orthopedist specializing in the foot and ankle.

The study was conducted in the Orthopedics and Traumatology Sector of a specialized hospital in Curitiba, Paraná, Brazil.

Through a retrospective analysis, we collected imaging tests from six patients diagnosed with tibial pilon fracture, including radiographs in the anteroposterior (AP) and lateral (L) views, computed tomography (CT) scans of the ankle, and 3D computed tomography reconstructions. No data regarding the patient or trauma was provided.

Supplementary tests for each patient included 4 groups: radiographs, CT scans, 3D reconstruction, and prototyping (3D printing). All 24 tests were randomized and numbered to avoid bias. Only the researchers knew which tests corresponded to the same patient.

The participants answered a questionnaire with images to classify the fractures according to the Rüedi-Allgöwer and AO/OTA systems and propose a treatment.

The interview occurred a single time after the participant signed an informed consent form, and there was no time limit for answering.

Initially, we assessed the interobserver agreement in each group (residents, orthopedists, and foot specialists) considering the four types of images (radiographs, CT, 3D CT, and 3D Model). Then, also for each medical group, we evaluated the agreement between the results of the four images. To assess the level of agreement among evaluators and image types, we estimated the Kappa agreement coefficients and their confidence intervals. Furthermore, we tested the significance of each Kappa coefficient and presented the *p* -values. The evaluation of the internal consistency of the questionnaire was carried out by estimating Cronbach alpha coefficients considering each group of evaluators and each image type. The bootstrap method (400 replications) was used to calculate 95% confidence intervals (95%CIs) for these coefficients. For the statistical tests, *p* -values < 0.05 were deemed significant, and for the intervals presented, we considered a 95% confidence level. Data were organized in an Excel (Microsoft Corp., Redmond, WA, United States) spreadsheet and analyzed using the IBM SPSS Statistics for Windows (IBM Corp., Armonk, NY, United States) software.

## Results

We analyzed the answers to the questionnaires about fracture classification and the proposed treatment considering radiographs, CT, 3D CT, and 3D models. We estimated Kappa agreement coefficients to assess the agreement level among residents, orthopedists, and foot specialists for each image (radiographs, CT, 3D CT, and 3D model). The tables present the percentages of agreement, the Kappa agreement coefficients (with 95% confidence intervals), and the *p* -values corresponding to the significance of the coefficients.

The agreement coefficient analysis used Landis and Koch interpretation of Kappa values. Kappa values above 0.8 indicate excellent agreement, while those ranging from 0.60 to 0.79 show substantial agreement, 0.40 to 0.59, moderate agreement, 0.20 to 0.39, low agreement, 0 to 0.19, poor agreement, and negative values indicate disagreement.

Interobserver agreement in the AO/OTA classification was low (k = 0.245) for radiographs but improved to moderate (k = 0.450) for the 3D model.

Interobserver agreement in the Rüedi-Allgöwer classification was moderate (k = 0.415) for radiographs but low for the 3D model (k = 0.329) ( Table 1 ).

**Table 1:** Agreement among residents, orthopedists, and specialists regarding imaging exams and 3D-printed models according to the AO/OTA classification

Image	Physicians	Agreement among physicians (%)	Kappa coefficient	95% confidence interval	*p* -value
**Radiograph**	General	43.9%	0.307	0–0.697	0.141
	Residents	47.0%	0.340	0–0.841	0.310
	Orthopedists	33.3%	0.176	0–0.576	0.211
	Foot specialists	38.9%	0.245	0–0.683	0.019
**Computed tomography**	General	31.3%	0.154	0.039–0.269	0.007
	Residents	24.4%	0.049	0.021–0.078	0.135
	Orthopedists	44.4%	0.256	0–0.625	0.055
	Foot specialists	50.0%	0.348	0–0.709	0.015
**3D computed tomography**	General	34.9%	0.208	0.060–0.356	0.062
	Residents	30.4%	0.158	0–0.327	0.087
	Orthopedists	41.7%	0.266	0–0.589	0.016
	Foot specialists	36.1%	0.200	0.055–0.345	0.063
**3D model**	General	43.6%	0.282	0–0.586	0.150
	Residents	41.1%	0.267	0–0.670	0.062
	Orthopedists	47.2%	0.306	0–0.634	0.017
	Foot specialists	63.9%	0.450	0.122–0.778	0.098

Abbreviations: 3D, tridimensional; AO/OTA, Arbeitsgemeinschaft für Osteosynthesefragen (AO, Association for the Study of Internal Fixation) Foundation/Orthopedic Trauma Association.

Interobserver agreement in the Rüedi-Allgöwer classification was moderate (k = 0.415) for radiographs but low for the 3D model (k = 0.329) ( Table 2 ).

**Table 2:** Agreement among residents, orthopedists, and specialists regarding imaging exams and 3D-printed models according to the Rüedi-Allgöwer classification

Image	Physicians	Agreement among physicians (%)	Kappa coefficient	95% confidence interval	*p* -value
**Radiograph**	General	61.3%	0.415	0–0.892	0.076
	Residents	57.1%	0.347	0–0.840	0.130
	Orthopedists	55.6%	0.323	0–0.955	0.247
	Foot specialists	72.2%	0.556	0–1	0.064
**Computed tomography**	General	52.2%	0.196	0–0.430	0.083
	Residents	48.8%	0.094	0–0.328	0.348
	Orthopedists	52.8%	0.186	0–0.685	0.383
	Foot specialists	69.4%	0.385	0–1	0.216
**3D computed tomography**	General	49.3%	0.204	0–0.432	0.070
	Residents	46.4%	0.176	0–0.40	0.100
	Orthopedists	50.0%	0.168	0–0.559	0.321
	Foot specialists	50.0%	0.127	0–0.587	0.509
**3D model**	General	56.8%	0.304	0.004–0.605	0.048
	Residents	58.3%	0.369	0–0.831	0.095
	Orthopedists	63.9%	0.329	0–0.841	0.159
	Foot specialists	66.7%	0.329	0–0.706	0.076

Abbreviation: 3D, tridimensional.

Interobserver agreement regarding management was poor (k = 0.168) for the 3D model and low for radiographs (k = 0.311) ( Table 3 ).

**Table 3:** Agreement among residents, orthopedists, and specialists regarding imaging exams and 3D-printed models according to the treatment proposed

Image	Physicians	Agreement among physicians (%)	Kappa coefficient	95% confidence interval	*p* -value
**Radiograph**	General	78.6%	0.311	0.216–0.406	< 0.001
	Residents	76.9%	0.233	0.115–0.351	< 0.001
	Orthopedists	79.9%	0.371	0.219–0.522	< 0.001
	Foot specialists	80.6%	0.390	0.212–0.569	< 0.001
**Computed tomography**	General	72.9%	0.181	0.109–0.254	< 0.001
	Residents	73.1%	0.133	0.063–0.203	< 0.001
	Orthopedists	72.5%	0.238	0.090–0.387	0.002
	Foot specialists	79.3%	0.393	0.227–0.558	< 0.001
**3D computed tomography**	General	74.0%	0.167	0.106–0.228	< 0.001
	Residents	72.9%	0.084	0.018–0.149	0.014
	Orthopedists	77.2%	0.271	0.157–0.384	< 0.001
	Foot specialists	73.2%	0.211	0.054–0.369	0.009
**3D model**	General	72.6%	0.168	0.091–0.246	< 0.001
	Residents	70.8%	0.093	0.001–0.186	0.048
	Orthopedists	76.5%	0.277	0.116–0.438	0.001
	Foot specialists	73.2%	0.234	0.083–0.386	0.003

Abbreviation: 3D, tridimensional.

A surprising finding was that CT images did not increase agreement for both classifications compared to plain radiographs. In the AO/OTA classification, residents presented a low agreement for radiographs (k = 0.340) and a poor agreement for CT (k = 0.049). Using the Rüedi-Allgöwer classification, foot specialists presented moderate agreement for radiographs (k = 0.556) but poor agreement for CT (k = 0.385), and low agreement for 3D CT reconstruction (k = 0.127).

## Discussion

A fracture classification system must be reliable, reproducible, logical, and clinically useful; its purpose is to help in clinical decision-making, facilitate communication among professionals, and enable comparisons in research. 17 18

In the present study, the Rüedi-Allgöwer and AO/OTA classification systems showed low to moderate agreement when using the 3D model. In the AO/OTA classification, agreement improved to moderate when experts evaluated the 3D model. Our hypothesis was corroborated with an increase in agreement only when 3D printing was evaluated by specialists in foot surgery using the AO/OTA classification. The other groups did not show statistical improvement. Byun et al. 19 evaluated the use of 3D CT regarding the same classifications, and they did not show an improved agreement between specialists and residents.

Computed tomography reportedly enables a better interpretation of the characteristics of each fracture and its joint fragments when compared with radiography. 17 20 21 However, the agreement with CT scans was not better than the one with radiographs in this study, which is consistent with similar studies, such as those by Ramappa et al., 22 Keiler et al., 23 and Martin et al. 20

The 3D model did not improve agreement regarding treatment recommendations, which is consistent with the study by Byun et al., 19 in which 3D CT did not improve agreement regarding treatment.

Keiler et al. 23 used the surgical procedure as a reference and evaluated the observers on the approach and implant positioning through 3D CT. These authors observed that the correlation improved significantly, especially in observers with less experience, suggesting that the 3D visualization of the injury may more beneficial for less-experienced surgeons.

## Conclusion

Tibial pilon fractures are complex joint injuries, usually secondary to high-energy trauma. The correct interpretation of the injury and subsequent management is essential to avoid sequelae for the patient. Imaging exams are critical for the surgeon's decision-making. Therefore, articles like the present have great value in maintaining scientific growth and improving orthopedic protocols.

In the current study, the use of the 3D model did not improve agreement regarding the treatment proposed by the groups for tibial pilon fractures. Concerning the classification systems, it only improved agreement in the AO/OTA classification when compared with the foot and ankle specialist group.

We also emphasize that the results obtained in the present study did not demonstrate a higher agreement with the use of CT scans compared with simple tibial pilon radiographs.

However, we should not rule out the potential use of 3D printing to interpret tibial pilon fractures. Further studies with larger sample sizes are required.
