# Supplementary material for: Use of Three-dimensional Printing for Tibial Pilon Fracture Diagnosis and Treatment
Source: Rev Bras Ortop (Sao Paulo). 2024 Jun 22;59(3):e456–61. doi: 10.1055/s-0044-1785514 (PMC11193579; doi:10.1055/s-0044-1785514)
Supplement: Related texts

# Research article: Uso da impressão tridimensional para auxílio diagnóstico e terapêutico das fraturas de pilão tibial

**Keywords:** fraturas da tíbia, fraturas ósseas, impressão tridimensional

## Abstract

**Objetivo** Avaliar se a impressão tridimensional (3D) aumenta a concordância na classificação de fraturas do pilão tibial.

**Métodos** Foram selecionadas radiografias, tomografias com reconstrução 3D e impressão de prototipagem em impressora 3D. Os exames foram apresentados a profissionais da área de Ortopedia e Traumatologia que classificaram as fraturas com base nas classificações da Arbeitsgemeinschaft für Osteosynthesefragen (AO, Associação para o Estudo da Fixação Interna) Foundation/Orthopedic Trauma Association (AO/OTA) e de Rüedi-Allgöwer. Posteriormente, os dados foram avaliados pelos coeficientes de concordância de Kappa.

**Resultados** O uso do modelo 3D não melhorou a concordância na fratura do pilão tibial quanto ao tratamento proposto pelos grupos. Em relação aos sistemas de classificação, somente a concordância na classificação AO/OTA melhorou quando foi utilizado o modelo 3D na avaliação pelos especialistas em pé e tornozelo.

**Conclusão** Apesar de o uso da impressão 3D ter relevância estatística para os cirurgiões especialistas em pé e tornozelo, ainda apresenta valores menores do que os ideais.

## Introdução

A fratura de pilão representa 1% das fraturas de membros inferiores, e de 5% a 10% das fraturas tibiais. 1 Além disso, 20% a 40% das fraturas são expostas. 2 É mais comum na quarta década de vida e em homens. 3 Ela geralmente ocorre como resultado de um trauma de alta energia com carga axial e força de cisalhamento, 4 como em quedas e em acidentes automobilísticos. 5 A fratura também pode ocorrer por trauma rotacional, como é o caso de lesões em atividades esportivas. 6 Neste caso, a fratura é decorrente de traumas de baixa energia e, consequentemente, causa menos danos aos tecidos moles e menor cominuição. A posição do pé no momento do impacto, juntamente com a direção e a amplitude da força, resulta em vários padrões de fratura e graus de cominuição. 7

Sistemas de classificação são úteis na prática clínica para avaliar a gravidade da lesão, guiar o tratamento cirúrgico, e facilitar a comunicação e a comparação para fins acadêmicos. As classificações mais utilizadas para as fraturas de pilão tibial são a de Rüedi-Allgöwer e a da Arbeitsgemeinschaft für Osteosynthesefragen (AO, Associação para o Estudo da Fixação Interna) Foundation/Orthopedic Trauma Association (AO/OTA). A classificação da AO/OTA leva em consideração a extensão do envolvimento articular e a cominuição, 8 e divide a fratura de pilão em 3 grupos: 43-A (fratura extra-articular), 43-B (fratura articular parcial) e 43-C (fratura articular completa). 9 Cada um desses grupos é dividido em três subgrupos, nos quais a complexidade aumenta e o prognóstico piora progressivamente. 6 Já a classificação de Rüedi-Allgöwer é dividida em três tipos, e leva em conta o grau de cominuição e o deslocamento articular. O tipo I é a fratura articular simples e sem desvio, o tipo II, a fratura articular simples, com desvio da superfície articular e sem cominuição, e tipo III, a fratura articular cominuta com desvio. 10

A indicação cirúrgica para a fixação das fraturas de pilão inclui fraturas expostas, com 2 mm de deslocamento articular, subluxação talar ou desalinhamento maior do que 5°. 10 Os objetivos principais do tratamento são proteger os tecidos moles, garantir o alinhamento adequado, restaurar a superfície articular 3 e permitir a reabilitação e a mobilização precoces. 10

A conduta e o tempo cirúrgico são determinados pelo estado de saúde geral do paciente, pela condição das partes moles adjacentes, pela cominuição da fratura e pela experiência do cirurgião. 11 Várias opções de tratamento são relatadas na literatura em caso de fratura de pilão tibial, incluindo redução aberta e fixação interna, fixação externa, e várias combinações e modificações dessas técnicas. 12 Em geral, a fixação externa é realizada primeiro para que haja a melhora dos danos às partes moles adjacentes, 13 o que diminui as taxas de infecção, deiscência e osteomielite. 12 Esse procedimento em dois estágios tem sido mundialmente popularizado no tratamento das fraturas de pilão tibial. 14 Por outro lado, quando a condição do tecido mole é boa, o tratamento por redução aberta e fixação interna já é inicialmente realizado. 15 A sequência geral no tratamento das fraturas de pilão tibial inclui o reestabelecimento do comprimento e do alinhamento, a restauração da superfície articular, o preenchimento dos defeitos metafisários e a reconexão à diáfise. 10

A impressão tridimensional (3D) é uma tecnologia de prototipagem rápida que usa um modelo digital 3D para construir um objeto. Tem se desenvolvido rapidamente e tem ganho boa visibilidade no meio ortopédico, pois o modelo impresso possibilita a visualização da fratura, permite que se faça um plano preciso e individual para os pacientes, assim como a simulação do procedimento, pois possibilita a visualização da anatomia da fratura antes do início da cirurgia, além de melhorar a comunicação entre médico e paciente. 16

O objetivo deste estudo é avaliar a concordância com relação às classificações da fratura de pilão tibial, assim como o tratamento proposto baseado nos exames de imagem, e comparar com a impressão 3D.

## Materiais e Métodos

Este trabalho obteve aprovação do Comitê de Ética, com registro na Plataforma Brasil sob o número do CAAE: 52795321.0.0000.5225.

Trata-se de um estudo observacional, transversal, retrospectivo, realizado com 16 profissionais da área de Ortopedia e Traumatologia, sendo eles: 8 médicos residentes, 4 médicos ortopedistas e 4 médicos ortopedistas especialistas em pé e tornozelo.

O estudo foi realizado no Setor de Ortopedia e Traumatologia de um hospital especializado da cidade de Curitiba, Paraná.

Por meio de análise retrospectiva, foram coletados os exames de imagem de seis pacientes com diagnóstico de fratura de pilão tibial, que tinham exames de radiografia nas incidências anteroposterior (AP) e de perfil (P), imagens de tomografia computadorizada (TC) do tornozelo, bem como reconstrução tomográfica 3D. Nenhum dado a respeito do paciente ou do trauma foi informado.

Os exames complementares de cada paciente foram divididos em 4 grupos: radiografias, cortes tomográficos, reconstrução 3D e prototipagem (impressão 3D). Todos os 24 exames foram randomizados e numerados a fim de evitar vieses. Somente os pesquisadores sabiam quais exames correspondiam ao mesmo paciente.

Os participantes responderam a um questionário que apresentava os exames de imagens e solicitava que eles classificassem as fraturas de acordo com as classificações de Rüedi-Allgöwer e da AO/OTA, assim como o tratamento proposto por eles.

A entrevista foi realizada apenas uma vez, somente após a obtenção do consentimento livre e esclarecido do participante, e não foi imposto limite de tempo.

Inicialmente, para cada grupo (residentes, ortopedistas e especialistas em pé), foi avaliada a concordância interobservador, considerando-se os quatro tipos de imagem (radiografia, TC, TC 3D e modelo 3D). Em seguida, também para cada grupo de formação médica, foi avaliada a concordância entre os resultados das quatro imagens. Para avaliar o nível de concordância entre avaliadores e entre tipos de imagem, foram estimados coeficientes de concordância de Kappa e apresentados os intervalos de confiança para esses coeficientes. Além disso, cada coeficiente de Kappa teve a sua significância testada, e foram apresentados o valores de *p* . A avaliação da consistência interna do questionário foi feita estimando-se coeficientes alfa de Cronbach e considerando cada grupo de avaliadores e cada tipo de imagem. Os intervalos de confiança de 95% (IC95%) para este coeficiente foram calculados considerando o método *bootstrap* (400 replicações). Para os testes estatísticos, valores de *p* < 0,05 indicaram significância, e para os intervalos apresentados, foi considerado o nível de confiança de 95%. Os dados foram organizados em planilha Excel (Microsoft Corp., Redmond, WA, Estados Unidos) e analisados com o programa IBM SPSS Statistics for Windows (IBM Corp., Armonk, NY, Estados Unidos).

## Resultados

A análise foi realizada com base nos questionários com perguntas sobre a classificação da fratura e o tratamento a ser escolhido considerando-se imagens de radiografia, TC, TC 3D e modelo 3D. Os coeficientes de concordância de Kappa foram estimados para avaliar a nível de concordância entre os grupos de residentes, ortopedistas, e especialistas em pé para cada uma das imagens. Nas tabelas a seguir são apresentados os percentuais de concordância, os coeficientes de concordância de Kappa (com intervalos de confiança de 95%) e os valores de *p* correspondentes à significância dos coeficientes.

A análise do coeficiente de concordância foi feita conforme a interpretação de Landis e Koch para valores de Kappa. Valores Kappa acima de 0,8 indicam excelente concordância, entre 0,60 e 0,79, concordância substancial, entre 0,40 e 0,59, concordância moderada, entre 0,20 e 0,39, concordância baixa, e entre 0 e 0,19, concordância ruim; valores negativos indicam discordância.

A concordância interobservador na classificação da AO/OTA foi baixa (k = 0,245) quando usada a radiografia, mas melhorou para moderada (k = 0,450) quando utilizado o modelo 3D na avaliação pelos especialistas ( Tabela 1 ).

**Tabela 1:** Concordância entre residentes, ortopedistas e especialistas em relação aos resultados de exames de imagem e impressão 3D segundo a classificação da AO/OTA

Imagem	Médicos	% de concordância entre os médicos	Coeficiente de Kappa	IC95%	*p*
**Radiografia**	Geral	43,9%	0,307	0–0,697	0,141
	Residentes	47,0%	0,340	0–0,841	0,310
	Ortopedistas	33,3%	0,176	0–0,576	0,211
	Especialistas em pé	38,9%	0,245	0–0,683	0,019
**TC**	Geral	31,3%	0,154	0,039–0,269	0,007
	Residentes	24,4%	0,049	0,021–0,078	0,135
	Ortopedistas	44,4%	0,256	0–0,625	0,055
	Especialistas em pé	50,0%	0,348	0–0,709	0,015
**TC 3D**	Geral	34,9%	0,208	0,060–0,356	0,062
	Residentes	30,4%	0,158	0–0,327	0,087
	Ortopedistas	41,7%	0,266	0–0,589	0,016
	Especialistas em pé	36,1%	0,200	0,055–0,345	0,063
**Modelo 3D**	Geral	43,6%	0,282	0–0,586	0,150
	Residentes	41,1%	0,267	0–0,670	0,062
	Ortopedistas	47,2%	0,306	0–0,634	0,017
	Especialistas em pé	63,9%	0,450	0,122–0,778	0,098

Abreviaturas: 3D, tridimensional; AO/OTA, Arbeitsgemeinschaft für Osteosynthesefragen (AO, Associação para o Estudo da Fixação Interna) Foundation/Orthopedic Trauma Association; IC95%, intervalo de confiança de 95%; TC, tomografia computadorizada.

A concordância interobservador na classificação de Rüedi-Allgöwer foi moderada (k = 0,415) quando utilizada a radiografia, mas, ao utilizar o modelo 3D, foi baixa (k = 0,329) ( Tabela 2 ).

**Tabela 2:** Concordância entre residentes, ortopedistas e especialistas em relação aos resultados de exames de imagem e impressão 3D segundo a classificação de Rüedi-Allgöwer

Imagem	Médicos	% de concordância entre os médicos	Coeficiente de Kappa	IC95%	*p*
**Radiografia**	Geral	61,3%	0,415	0–0,892	0,076
	Residentes	57,1%	0,347	0–0,840	0,130
	Ortopedistas	55,6%	0,323	0–0,955	0,247
	Especialistas em pé	72,2%	0,556	0–1	0,064
**TC**	Geral	52,2%	0,196	0–0,430	0,083
	Residentes	48,8%	0,094	0–0,328	0,348
	Ortopedistas	52,8%	0,186	0–0,685	0,383
	Especialistas em pé	69,4%	0,385	0–1	0,216
**TC 3D**	Geral	49,3%	0,204	0–0,432	0,070
	Residentes	46,4%	0,176	0–0,40	0,100
	Ortopedistas	50,0%	0,168	0–0,559	0,321
	Especialistas em pé	50,0%	0,127	0–0,587	0,509
**Modelo 3D**	Geral	56,8%	0,304	0,004–0,605	0,048
	Residentes	58,3%	0,369	0–0,831	0,095
	Ortopedistas	63,9%	0,329	0–0,841	0,159
	Especialistas em pé	66,7%	0,329	0–0,706	0,076

Abreviaturas: 3D, tridimensional; IC95%, intervalo de confiança de 95%; TC, tomografia computadorizada.

A concordância interobservador quanto à conduta foi ruim (k = 0,168) quando utilizado o modelo 3D; já com a radiografia, foi baixa (k = 0,311) ( Tabela 3 ).

**Tabela 3:** Concordância entre residentes, ortopedistas e especialistas em relação aos resultados de exames de imagem e impressão 3D segundo a conduta

Imagem	Médicos	% de concordância entre os médicos	Coeficiente de Kappa	IC95%	*p*
**Radiografia**	Geral	78,6%	0,311	0,216–0,406	< 0,001
	Residentes	76,9%	0,233	0,115–0,351	< 0,001
	Ortopedistas	79,9%	0,371	0,219–0,522	< 0,001
	Especialistas em pé	80,6%	0,390	0,212–0,569	< 0,001
**TC**	Geral	72,9%	0,181	0,109–0,254	< 0,001
	Residentes	73,1%	0,133	0,063–0,203	< 0,001
	Ortopedistas	72,5%	0,238	0,090–0,387	0,002
	Especialistas em pé	79,3%	0,393	0,227–0,558	< 0,001
**TC 3D**	Geral	74,0%	0,167	0,106–0,228	< 0,001
	Residentes	72,9%	0,084	0,018–0,149	0,014
	Ortopedistas	77,2%	0,271	0,157–0,384	< 0,001
	Especialistas em pé	73,2%	0,211	0,054–0,369	0,009
**Modelo 3D**	Geral	72,6%	0,168	0,091–0,246	< 0,001
	Residentes	70,8%	0,093	0,001–0,186	0,048
	Ortopedistas	76,5%	0,277	0,116–0,438	0,001
	Especialistas em pé	73,2%	0,234	0,083–0,386	0,003

Abreviaturas: 3D, tridimensional; IC95%, intervalo de confiança de 95%; TC, tomografia computadorizada.

Um dado que nos surpreendeu foi que as imagens tomográficas não aumentaram a concordância para ambas as classificações quando comparados a exames de radiografia simples. Quando foi usada a classificação da AO/OTA, os residentes tiveram concordância baixa com radiografia (k = 0,340), que caiu para concordância ruim com a TC (k = 0,049). Já quando foi utilizada a classificação de Rüedi-Allgöwer, os especialistas em pé tiveram uma concordância moderada com a radiografia (k = 0,556), mas tiveram concordância ruim quando avaliadas imagens de TC (k = 0,385), e concordância baixa quanto às imagens de TC 3D (k = 127).

## Discussão

Um sistema de classificação de fratura deve ser confiável, reprodutível, lógico e útil clinicamente; seu propósito é o de ajudar na tomada de decisão clínica, facilitar comunicação entre profissionais e permitir comparações em pesquisas. 17 18

Neste estudo, os sistemas de classificação de Rüedi-Allgöwer e da AO/OTA mostraram concordância de baixa a moderada quando utilizado o modelo 3D. Na classificação da AO/OTA, a concordância melhorou para moderada quando o modelo 3D era avaliado por especialistas. A hipótese levantada por nós foi corroborada com um aumento da concordância somente quando o modelo 3D foi avaliado por especialistas em cirurgia do pé fazendo uso da classificação da AO/OTA. Os demais grupos não apresentaram melhora estatística. O trabalho Byun et al. 19 avaliou o uso da TC 3D com relação às mesmas classificações, e não se observou melhora da concordância entre especialistas e residentes.

Espera-se que a TC permita uma melhor interpretação da característica de cada fratura, assim como de seus fragmentos articulares, quando comparada à radiografia. 17 20 21 No entanto, a concordância com a TC não foi melhor em comparação com a das radiografias neste estudo, o que corrobora outros trabalhos similares, como os de Ramappa et al., 22 Keiler et al. 23 e de Martin et al. 20

Neste estudo, o modelo 3D não melhorou a concordância das recomendações de tratamento, assim como no estudo de Byun et al., 19 em que a TC 3D não melhorou a concordância com o tratamento.

Keiler et.al. 23 utilizaram o procedimento cirúrgico realizado como referência, e avaliaram os observadores quanto à via de acesso e à posição do implante por meio da TC 3D; os autores observaram que a correlação melhorou significativamente, principalmente entre os observadores com menos experiência, o que sugere que a visão 3D da fratura pode beneficiar mais os cirurgiões com menos experiência.

## Conclusão

As fraturas do pilão tibial são lesões articulares complexas, geralmente secundárias a um trauma de alta energia. A correta interpretação da lesão e posterior conduta são imprescindíveis para evitar sequelas ao paciente. Os exames de imagem são fundamentais para a tomada de decisão do cirurgião. Portanto, artigos como este são de grande valia para a manutenção do crescimento científico e para a aprimoração dos protocolos ortopédicos.

Neste trabalho, o uso do modelo 3D não melhorou a concordância quanto ao tratamento proposto pelos grupos para a fratura do pilão tibial em relação aos sistemas de classificação; somente a concordância na classificação AO/OTA melhorou quando comparado o grupo de especialistas em pé e tornozelo.

Ressaltamos ainda que os resultados obtidos neste artigo não demonstraram maior concordância com o uso da TC quando comparada a radiografia simples de pilão tibial.

No entanto, não se deve descartar a possibilidade de adesão à impressão 3D para a interpretação das fraturas de pilão tibial. É necessário abranger o trabalho para um número amostral ainda maior.
